# Association of Dermatological Manifestations in Patients With Type 2 Diabetes Mellitus With Respect to Duration of Diabetes

**DOI:** 10.7759/cureus.88681

**Published:** 2025-07-24

**Authors:** Syeda Shahmoona Tirmizi, Maniha Zulfiqar, Esha Moosa, Rahim Rasool, Sarina Shami, Areebah Alam, Adnan Anwar, Hassan Nadeem, Atif A Hashmi

**Affiliations:** 1 Dermatology, Hamdard College of Medicine and Dentistry, Karachi, PAK; 2 Internal Medicine, Liaquat National Hospital and Medical College, Karachi, PAK; 3 Internal Medicine, Ziauddin University, Karachi, PAK; 4 Internal Medicine, Jinnah Sindh Medical University, Karachi, PAK; 5 Physiology, Hamdard College of Medicine and Dentistry, Karachi, PAK; 6 Internal Medicine, Sindh Government Hospital, Karachi, PAK; 7 Internal Medicine, Quaid-e-Azam Medical College, Bahawalpur, Bahawalpur, PAK; 8 Pathology, Liaquat National Hospital and Medical College, Karachi, PAK

**Keywords:** dermatological manifestations, diabetes, duration, type 2 diabetes mellitus, xerosis

## Abstract

Objective

Diabetes mellitus is a disease that can affect various organs, and so all the symptoms should be investigated. Nonetheless, skin symptoms have received less attention than others. The duration of diabetes plays a critical role in the development and progression of systemic and dermatological complications. Existing evidence suggests that many cutaneous complications, such as diabetic dermopathy, xerosis, and infections, tend to increase in frequency and severity with longer disease duration. Therefore, this study assessed and compared the demographic, clinical, lifestyle, and dermatological features of patients with type 2 diabetes mellitus (T2DM) based on the duration of the disease.

Methodology

This cross-sectional study was performed at multiple secondary care hospitals and primary care centers. The time period of the study was from December 1, 2024, to May 30, 2025. This study included 290 patients with confirmed T2DM, categorized into three groups based on diabetes duration: Group A (less than one year), Group B (one to five years), and Group C (more than five years). Data were collected through clinical examination and structured questionnaires covering demographic details, lifestyle factors, treatment modalities, physiological parameters, and dermatological findings. Statistical analysis was applied using the chi-square test and a Kruskal-Wallis test, with p < 0.05 reflecting statistically significant.

Results

The comparison of three groups showed significant differences in gender, socioeconomic status, dyslipidemia, smoking history, and diabetes treatment. Group A had more individuals from a high socioeconomic background, while Group C had the most from the middle class. Dermatological conditions such as acanthosis nigricans (p < 0.001), callosities (p = 0.020), and bullae (p = 0.027) were significantly associated with longer diabetes duration.

Conclusion

This study concluded that the duration of T2DM patients with a longer diabetes duration was linked to lower BMI, better glycemic control, and more lifestyle-based treatments, while shorter duration was associated with higher age, BMI, dyslipidemia, and smoking. Dermatological manifestations such as acanthosis nigricans, callosities, and bullae indicated a significant association with diabetes duration.

## Introduction

Diabetes mellitus is a significant global health issue due to its rising prevalence and the wide range of systemic and localized complications it causes. Currently, the condition affects over half a billion people worldwide, impacting over 10.5% of the adult population. Among its many manifestations, skin-related symptoms are particularly common, highlighting the crucial role of dermatologists in recognizing these signs. Skin disorders, for instance, acanthosis nigricans (AN), necrobiosis lipoidica, diabetic dermopathy (DD), bacterial and fungal infections, and xerosis often accompany diabetes and may even appear before the disease is properly diagnosed [1,2].

Skin conditions are expected to affect approximately 79.2% of individuals with diabetes. In a study involving 750 diabetic patients, the most frequently reported skin issues were cutaneous infections (47.5%), xerosis (26.4%), and inflammatory skin disorders (20.7%). People with type 2 diabetes mellitus (T2DM) are generally more susceptible to developing these skin manifestations as compared with type 1 diabetes mellitus (T1DM) [3].

Dermatological manifestations in diabetes mellitus range from minor cosmetic issues to serious, potentially life-threatening disorders. Identifying these skin changes can offer important insights into a patient's metabolic status, assist in diagnosing diabetes, and serve as indicators for monitoring glycemic control. AN is a well-known skin disorder associated with diabetes, commonly seen in both sexes across all age groups. Their prevalence is higher in individuals with T2DM [4] and those with darker skin tones, particularly individuals of African American, Hispanic, or Native American descent [5]. Another relatively rare condition, bullosis diabeticorum (BD), is characterized by the sudden formation of blisters and is most frequently observed in individuals with long-standing diabetes, often in conjunction with complications such as neuropathy, nephropathy, and retinopathy [6,7].

Xerosis, or abnormally dry skin, is among the most frequently observed dermatological conditions in individuals with diabetes, affecting up to 40% of this population [8]. It typically appears as scaling, cracking, or a rough skin texture, most commonly on the feet. The severity of xerosis may be greater in obese diabetic patients due to increased hypohidrosis (reduced sweating) of the feet [9]. This condition is often linked to microvascular complications in diabetes [10]. To prevent further issues, such as skin fissures or secondary infections, xerosis should be treated effectively with moisturizing agents, such as ammonium lactate-based emollients [11].

T2DM is a chronic condition related to many systemic and dermatological complications that may vary with the duration of the disease. Understanding these changes is crucial for early intervention and improved patient care. Given that the study was conducted in Karachi, a densely populated metropolitan area with a unique ethnic and socioeconomic status, it is important to recognize that regional population specifics, such as lifestyle patterns, healthcare access, and environmental factors, may have influenced the observed outcomes. Therefore, this study aims to identify the prevalence patterns and assess the association of various dermatological manifestations with the duration of T2DM, in order to explore potential indicators for early clinical recognition and management of diabetes-related skin complications.

## Materials and methods

This cross-sectional study was performed at multiple secondary care hospitals and primary care centers. A non-probability convenience sampling technique was applied. Ethical approval was obtained from the Institutional Review Board of Sindh Government Hospital, Karachi, Pakistan (Approval No. SGH (2A/Landhi) 1739). The time period of the study was from December 1, 2024, to May 30, 2025. Using OpenEpi software (version 3.0.1; Dean AG, Sullivan KM, Soe MM. OpenEpi: Open Source Epidemiologic Statistics for Public Health) for sample size calculation, the prevalence of issues related to skin in T2DM was 79.2%, based on a previously published study [2]. The estimated sample size was 254 patients. The study involved 290 patients aged 18 years or older diagnosed with T2DM. Participants were categorized into three groups based on the duration of diabetes: Group A (less than one year), Group B (one to five years), and Group C (more than five years). These duration brackets were selected to reflect the progressive nature of T2DM and its complications [12]. Participants were included if they had no recent history of hospitalization, no active infections, and were not undergoing treatment for any major systemic illnesses unrelated to diabetes that could interfere with dermatological evaluation. Participants were excluded with T1DM, those with uncontrolled systemic illnesses (e.g., renal failure, malignancies), current or recent infections, or pre-existing dermatological conditions unrelated to diabetes that could confound the assessment.

Informed consent was obtained from all participants prior to data collection. Detailed clinical evaluations were performed to monitor glycemic control and identify diabetes-related complications. Glycemic status was primarily assessed through glycosylated hemoglobin (HbA1c) levels, reflecting long-term blood sugar management, and postprandial blood glucose levels measured two hours after meals to assess glucose metabolism. Cardiovascular complications were evaluated using physical parameters, including blood pressure and heart rate assessments. Data were collected using a structured questionnaire and clinical examination, focusing on demographic details, lifestyle habits, clinical parameters, and dermatological manifestations (Appendix A). Anthropometric measurements (age, weight, height, BMI), physiological parameters (blood pressure, respiratory rate, heart rate, random blood sugar), and treatment modalities were recorded. Dyslipidemia was identified through analysis of lipid profile results. All participants underwent comprehensive dermatological examinations aimed at detecting skin conditions frequently associated with T2DM. These evaluations were conducted by trained clinicians under standardized conditions. While the assessments were not blinded, evaluators received prior training to ensure consistency and minimize observer bias. Dermatological findings were recorded using a predefined checklist and standardized diagnostic criteria; however, confirmation by a board-certified dermatologist was not employed, representing a methodological limitation. Dermatological manifestations are defined as any observable or clinically diagnosed skin, hair, or nail abnormalities that are known to be associated with T2DM. These may arise due to metabolic, vascular, neuropathic, or immunologic complications of diabetes [13]. Key dermatological manifestations assessed included xerosis, ichthyosis, diabetic rubeosis, AN, callosities, and bullae, as mentioned in Appendix B.

Data were entered and analyzed using IBM SPSS Statistics for Windows, Version 20 (Released 2012; IBM Corp., Armonk, New York, United States). The demographic information and dermatological manifestations associated with T2DM were presented as frequencies and percentages. Quantitative variables were documented as means and standard deviations. A chi-square test was applied to observe the association of dermatological features in T2DM. Additionally, a Kruskal-Wallis test was applied to determine the association among the means of demographic variables. A p-value of less than 0.05 was considered statistically significant.

## Results

This study included 290 patients with T2DM with respect to the duration of diabetes, dividing them into three groups: Group A (less than one year), Group B (one to five years), and Group C (more than five years). The mean age was significantly greater in Group A (60.38 ± 16.03 years) compared to Group B (52.73 ± 14.32 years) and Group C (55.51 ± 14.44 years) (p < 0.001). Similarly, Group A had the highest mean weight (72.0 ± 13.11 kg), followed by Group B (67.06 ± 15.26 kg) and Group C (66.42 ± 14.94 kg), with a statistically significant association (p < 0.001). Height also varied significantly among the groups (p = 0.004), with Group C having the tallest average height (69.07 ± 10.41 inches). The mean BMI was highest in Group A (26.06 ± 11.42 kg/m^2^) and Group B (26.02 ± 9.97 kg/m^2^), while Group C had a lower mean BMI (23.54 ± 11.63 kg/m^2^) (p = 0.007). Respiratory rate varied significantly (p < 0.001), with Groups A and C showing higher rates (20.46 ± 6.03 and 20.23 ± 5.84 cycles/min, respectively) compared to Group B (17.43 ± 5.19 cycles/min). Blood pressure was insignificantly associated with the diabetes duration (p = 0.187). However, the duration of hypertension exhibited a significant association among the groups (p = 0.014), with the longest duration reported in Group A (5.85 ± 5.98 years). Heart rate was significantly higher in Group A (87.82 ± 10.51 beats/min) and Group C (88.16 ± 11.23 beats/min) than in Group B (81.2 ± 11.55 beats/min) (p < 0.001). Lastly, random blood sugar levels were significantly elevated in Groups A (337.37 ± 97.8 mg/dL) and B (323.40 ± 108.99 mg/dL) compared to Group C (229.8 ± 90.91 mg/dL) (p < 0.001), as depicted in Table 1.

**Table 1:** Demographic details of patients with T2DM based on duration of diabetes (n = 290) T2DM: type 2 diabetes mellitus; BMI: body mass index

Variables	Group A (<1 year) (mean ± SD)	Group B (1 to 5 years) (mean ± SD)	Group C (>5 years) (mean ± SD)	p-value
Age (years)	60.38 ± 16.03	52.73 ± 14.32	55.51 ± 14.44	<0.001
Weight (kg)	72.0 ± 13.11	67.06 ± 15.26	66.42 ± 14.94	<0.001
Height (inch)	68.25 ± 10.82	64.84 ± 8.19	69.07 ± 10.41	0.004
BMI (kg/m^2^)	26.06 ± 11.42	26.02 ± 9.97	23.54 ± 11.63	0.007
Respiratory rate (cycles/min)	20.46 ± 6.03	17.43 ± 5.19	20.23 ± 5.84	<0.001
Blood pressure (mmHg)	179.73 ± 43.98	173.50 ± 48.29	178.42 ± 49.65	0.187
Duration of hypertension (years)	5.85 ± 5.98	4.27 ± 4.16	5.0 ± 3.30	0.014
Heart rate (beats/min)	87.82 ± 10.51	81.2 ± 11.55	88.16 ± 11.23	<0.001
Random blood sugar (RBS) (mg/dL)	337.37 ± 97.8	323.40 ± 108.99	229.8 ± 90.91	<0.001

A comparison of demographic, lifestyle, and clinical characteristics among three groups revealed a highly substantial association in gender distribution across the groups (p < 0.001), with more than half in Groups A and C being males, compared with Group B. Socioeconomic status also varied significantly (p = 0.006); Group C had the highest proportion of participants from the middle socioeconomic class (93, 71.5%), whereas Group A had a larger proportion from the high socioeconomic class (42, 32.3%). An insignificant association was observed in the history of hypertension among the groups (p = 0.920), with roughly 91 (70.0%) individuals in each group reporting a positive history. However, dyslipidemia was significantly more common in Groups A and B (98, 75.4%) and (106, 81.5%), respectively, compared to Group C (79, 60.8%) (p=0.001). A significant variation was also observed in smoking history (p < 0.001); Group A had the highest proportion of smokers (60, 46.2%), while Group B had the lowest (21, 16.2%). Regarding diabetes therapy, a statistically significant difference was seen in the types of treatments used across the groups (p < 0.001), as depicted in Table 2.

**Table 2:** Comparison of demographic, lifestyle, and clinical characteristics based on duration of diabetes

Variables		Group A, n (%)	Group B, n (%)	Group C, n (%)	Pearson chi-square	p-value
Gender	Male	76 (58.5)	36 (27.7)	83 (63.8)	39.569	<0.001
	Female	54 (41.5)	94 (72.3)	47 (36.2)		
Socioeconomic status	Low	22 (16.9)	23 (17.7)	15 (11.5)	14.33	0.006
	Middle	66 (50.8)	82 (63.1)	93 (71.5)		
	High	42 (32.3)	25 (19.2)	22 (16.9)		
History of hypertension	Yes	90 (69.2)	88 (67.7)	91 (70.0)	0.168	0.920
	No	40 (30.8)	42 (32.3)	39 (30.0)		
Dyslipidemia	Yes	98 (75.4)	106 (81.5)	79 (60.8)	14.86	0.001
	No	74 (24.6)	24 (18.5)	51 (39.2)		
History of smoking	Yes	60 (46.2)	21 (16.2)	42 (32.3)	27.14	<0.001
	No	70 (53.8)	109 (83.8)	88 (67.7)		
Therapy used	Oral hypoglycemic drugs	59 (45.4)	62 (47.7)	31 (23.8)	43.15	<0.001
	Insulin	42 (32.3)	30 (23.1)	27 (20.8)		
	Diet only	8 (6.2)	11 (8.5)	29 (22.3)		
	Diet along with oral hypoglycemic drugs	13 (10.0)	12 (9.2)	28 (21.5)		
	Diet along with insulin	8 (6.2)	15 (11.5)	15 (11.5)		

**Figure 1:**
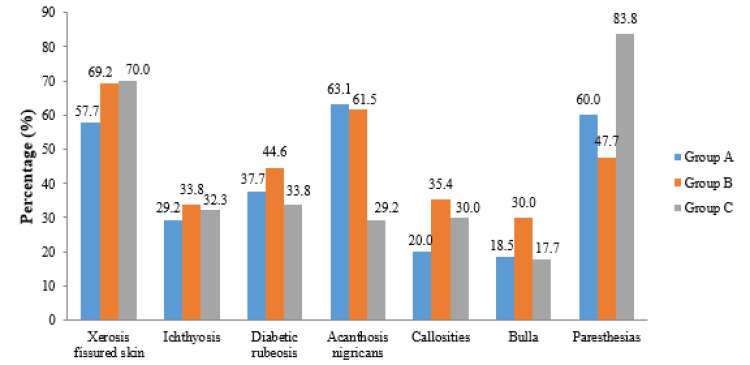
Graphical presentation of distribution of dermatological symptoms in patients with T2DM with duration of diabetes T2DM: type 2 diabetes mellitus

The distribution of dermatological symptoms among individuals with T2DM revealed that xerosis with fissured skin was more commonly observed in Groups B and C (90, 69.2%) and (91, 70.0%), respectively, compared to Group A (75, 57.7%), but the statistically insignificant (p = 0.065). Similarly, the prevalence of ichthyosis and diabetic rubeosis did not significantly differ among the groups (p = 0.718 and p = 0.196, respectively). In contrast, a highly significant difference was found in the occurrence of AN (p < 0.001), which was most prevalent in Groups A and B (82, 63.1%) and (80, 61.5%), and significantly lower in Group C (38, 29.2%). Callosities were also significantly more frequent in Group B (46, 35.4%) compared to Group A (26, 20.0%) and Group C (39, 30.0%) (p = 0.020). The presence of bullae showed a statistically significant variation (p = 0.027), with the highest frequency in Group B (39, 30.0%), as depicted in Table 3 and Figure 1.

**Table 3:** Dermatological symptoms in patients with T2DM with duration of diabetes T2DM: type 2 diabetes mellitus

Variables			Group A, n (%)	Group B, n (%)	Group C, n (%)	Pearson chi-square	p-value
Xerosis fissured skin	Yes	75 (57.7)		90 (69.2)	91 (70.0)	5.48	0.065
	No	55 (42.3)		40 (30.8)	39 (30.0)		
Ichthyosis	Yes	38 (29.2)		44 (33.8)	42 (32.3)	0.662	0.718
	No	92 (70.8)		86 (66.2)	88 (67.7)		
Diabetic rubeosis	Yes	49 (37.7)		58 (44.6)	44 (33.8)	3.26	0.196
	No	81 (62.3)		72 (55.4)	86 (66.2)		
Acanthosis nigricans	Yes	82 (63.1)		80 (61.5)	38 (29.2)	38.01	<0.001
	No	48 (36.9)		50 (38.5)	92 (70.8)		
Callosities	Yes	26 (20.0)		46 (35.4)	39 (30.0)	7.78	0.020
	No	104 (80.0)		84 (64.6)	91 (70.0)		
Bulla	Yes	24 (18.5)		39 (30.0)	23 (17.7)	7.19	0.027
	No	106 (81.5)		91 (70.0)	107 (82.3)		

## Discussion

Diabetes mellitus is the most common endocrine disorder that has an impact on the skin. Numerous skin conditions are linked to this disease. In the present study, xerosis with fissured skin was prevalent across all groups, particularly in Groups B and C (69.2% and 70.0%), though the difference did not reach statistical significance (p = 0.065). These findings align with those reported by Vâță et al., who observed xerosis in 45% of individuals with diabetes [14], and by Sabah Abdulridha Budair et al., who also identified xerosis as one of the most frequently occurring skin manifestations in diabetic patients [15]. In contrast, Sani et al. and Garg et al. found lower rates (10-19%), possibly due to geographical or methodological differences [16,17]. The rising occurrence of skin involvement in diabetic patients with increasing age, disease duration, and severity may be due to the body's reduced resistance, prolonged duration of diabetes, and inadequate blood sugar control.

The present study showed that ichthyosis was observed to be uniformly distributed in all groups, although not significantly different among our study groups (p = 0.718). While not frequently reported in the comparative studies, Sani et al. mentioned idiopathic guttate hypomelanosis as a common finding; it was observed in 61% of patients, while infections of bacterial, fungal, and viral origin were observed in 30%. Additional skin conditions included DD, which was present in 17% of patients [16].

Similarly, the present study observed that AN showed a highly significant variation (p < 0.001), predominantly affecting Groups A (82, 63.1%) and B (80, 61.5%). This supports its recognized association with insulin resistance and metabolic dysfunction. These findings were inconsistent with other studies, which reported AN prevalence between 18% and 19% [15,17,18].

Likewise, a prospective observational study involving 320 diabetic patients (174 males and 146 females) found that 91.2% exhibited some form of skin manifestation. These were significantly more prevalent in patients with T2DM (98.2%) compared to those with T1DM (34.3%) (p < 0.001). Additionally, patients with a diabetes duration of over five years had a greater prevalence (98%) of skin conditions than those with less than five years (80.6%), which was also statistically significant (p < 0.001). Specific conditions, such as gangrene, DD, diabetic foot, bullae, and fungal infections, showed significant differences based on disease duration (p < 0.05) [19]. Callosities were significantly more common in Group B (46, 35.4%) than in Group A (26, 20.0%) and Group C (39, 30.0%) (p = 0.020). The presence of bullae also varied significantly, being most frequent in Group B (39, 30.0%) (p = 0.027).

AN exhibited a statistically significant association across groups (p < 0.001), being more common in Group A (63.1%) and Group B (61.5%) than in Group C (29.2%). This condition is well-established in the literature as an indicator of insulin resistance and a potential indicator of increased macrovascular risk. The prevalence of AN varies by ethnicity, with rates reported between 1% and 5% in individuals of Caucasian descent and up to 13% among those of Black African and Hispanic populations [20,21]. Additionally, recent research has identified AN as an independent cardiovascular risk factor, especially among obese adolescents [22]. In a study by Trihan et al., DD was found to be the most frequent skin manifestation in patients with T2DM, affecting 17.8% (38 out of 213) of participants [23].

This cross-sectional, single-center study has several limitations that affect the strength and generalizability of its findings. The design prevents causal inferences, and the use of convenience sampling introduces potential selection bias. Reliance on self-reported lifestyle data may result in recall bias, and the lack of multivariate analysis limits the control of confounding variables. Additionally, the absence of longitudinal follow-up restricts understanding of disease progression, and dermatological assessments may be subject to observer bias due to a lack of blinding. A more robust methodological approach incorporating standardized diagnostic tools, multivariate analysis, and a multicenter design would enhance future research. Nonetheless, the study contributes valuable insight into the dermatological aspects of T2DM, an often overlooked area in clinical care.

## Conclusions

This study concluded that the duration of T2DM significantly impacts various demographic, clinical, and dermatological characteristics. Patients with a longer diabetes duration were linked to lower BMI, better glycemic control, and more lifestyle-based treatments, while shorter duration was associated with higher age, BMI, dyslipidemia, and smoking. Dermatological manifestations such as AN, callosities, and bullae showed a significant association with diabetes duration. Interestingly, the correlation between longer diabetes duration and better metabolic indicators may appear counterintuitive. Possible explanations include survivorship bias, more intensive treatment over time, or improved self-care behaviors among patients with longer disease duration. To enhance scientific rigor, future studies should adopt longitudinal or interventional designs, incorporate glycemic control parameters and comorbid conditions more systematically, and utilize analytical models to control for confounders.

## Appendices

Appendix A 

**Table 4:** Section A: Demographic and clinical information

No.	Question	Response
1	Age	________ years
2	Weight	________ kg
3	Height	________ inches
4	Do you have a history of dyslipidemia (high cholesterol/triglycerides)?	☐ Yes ☐ No
5	Do you have a history of hypertension (high blood pressure)?	☐ Yes ☐ No
6	If yes, how long have you had high blood pressure?	________ years
7	Do you have Type 2 Diabetes Mellitus?	☐ Yes ☐ No
8	How long have you had diabetes?	☐ Less than 1 year ☐ 1–5 years ☐ More than 5 years
9	What diabetes treatment are you currently using?	☐ Oral hypoglycemics ☐Insulin ☐ Diet only ☐ Diet + Oral hypoglycemics ☐ Diet + Insulin
10	Socioeconomic Status	☐ Low ☐ Middle ☐ High
11	Gender	☐ Male ☐ Female
12	Do you smoke or have a history of smoking?	☐ Yes ☐ No
13	Current Blood Pressure (if known)	________ mmHg
14	Current Heart Rate (if known)	________ beats/min
15	Respiratory Rate (if known)	________ breaths/min
16	Recent Random Blood Sugar (if known)	________ mg/dL

Appendix B 

**Table 5:** Section B: Dermatological manifestations Have you experienced any of the following skin-related symptoms in the past 6 months?

No.	Symptom	Response
17	Xerosis (dry, cracked, or fissured skin)	☐ Yes ☐ No
18	Ichthyosis (scaly skin)	☐ Yes ☐ No
19	Diabetic rubeosis (reddish skin discoloration, especially on the face)	☐ Yes ☐ No
20	Acanthosis nigricans (dark, velvety patches, usually in folds like neck or armpits)	☐ Yes ☐ No
21	Callosities (thickened skin or calluses on feet)	☐ Yes ☐ No
22	Bullae (fluid-filled blisters)	☐ Yes ☐ No
